# VDR and gemini ligands

**DOI:** 10.18632/oncotarget.5446

**Published:** 2015-09-01

**Authors:** Gilles Laverny, Daniel Metzger

**Affiliations:** Department of Functional Genomics and Cancer, Institut de Génétique et de Biologie Moléculaire et Cellulaire (IGBMC), Institut National de Santé et de Recherche Médicale (INSERM) U964/Centre National de Recherche Scientifique (CNRS) UMR 7104/Université de Strasbourg, Illkirch, France

**Keywords:** vitamin D, nuclear receptor, repression

The active form of vitamin D, 1α,25-dihydroxyvitamin D3 [1,25(OH)_2_D_3_; calcitriol], plays a key role in mineral and bone homeostasis, and exerts potent anti-inflammatory and anti-proliferative activities [1]. It is thus a potential pharmacological agent to treat various diseases, including autoimmune disorders, infections and cancer [1]. However, the 1,25(OH)_2_D_3_ doses required to elicit such effects induce hypercalcemia, resulting in ectopic calcification of the vascular wall, kidney and other soft tissues, that can lead to organ failure and death [1]. 1,25(OH)_2_D_3_ activities are mediated by the Vitamin D receptor (VDR; NR1I1), a member of the nuclear receptor superfamily [1]. More than 3000 VDR ligands were synthesized using medicinal chemistry approaches, but all those exhibiting potent anti-inflammatory and/or anti-proliferative properties still have hypercalcemic activities, which limit their clinical use [1].

VDR loss-of-function mutations in humans, termed Vitamin D-Dependent Rickets type-II (VDDR-II), and VDR-null mice develop skeletal deformities, osteomalacia, hypocalcemia and hypophosphatemia [2]. In addition, VDR DNA-binding deficient patients and VDR-null mice display alopecia [2]. Hair follicle defects in VDR-null mice are prevented by transgenic expression of ligand-binding deficient VDR in keratinocytes [3]. In contrast, severe deficiencies in 1,25(OH)_2_D_3_ induced by dysfunctional 25(OH)-vitaminD_3_-1α-hydroxylase (Cyp27b1), the enzyme that converts 25(OH)D_3_ to 1,25(OH)_2_D_3_ in VDDR-I patients, as well as in Cyp27b1-null mice, do not induce alopecia, even though they lead to rickets, which can be treated by 1,25(OH)_2_D_3_ [2, 4]. Similarly, patients and mice expressing a mutated VDR with reduced affinity for 1,25(OH)_2_D_3_ without impairing DNA binding have skeletal but no hair defects [2]. Thus, it appears that liganded VDR is essential for mineral ion homeostasis and skeletal metabolism, whereas ligand-independent VDR activities control hair cycling.

Phenotypic analyses of mutant mice suggested that bone and mineral homeostasis alterations might be more pronounced in Cyp27b1-null mice than in VDR-null [4, 5]. Moreover, data presented in a recent report indicated that the size of mice expressing VDR^L233S^, a VDR bearing a mutation in the ligand binding domain that impairs calcitriol binding, was reduced compared to VDR-null [3]. Taken together, these results indicated that unliganded VDR might induce more severe skeletal defects than VDR deficiency.

To characterise VDR ligand-independent activities, we generated, based on VDR structural analyses, mice expressing a ligand binding domain point-mutated VDR (VDRgem) that is unresponsive to 1,25(OH)_2_D_3_ [6]. Phenotypic analyses showed that mineral ion and bone homeostasis was more impaired in VDRgem than in VDR-null mice, even though they had no hair defects [6]. Moreover, selective ablation of VDR in intestinal epithelial cells did not affect mineral ion homeostasis [7], while selective expression of VDRgem in such cells induced hypocalcemia (our unpublished data), thus demonstrating that intestinal unliganded VDR strongly impairs calcium homeostasis. Importantly, our study revealed that apo-VDRgem binds to VDR response elements of VDR target genes in the mouse duodenum and represses many genes [6]. Therefore, the repressive activity of unliganded VDR in intestinal epithelial cells accounts, at least in part, for increased bone and mineral defects, compared to VDR deficiency.

Alopecia in VDDR-II patients is thought to be associated with the severity of rickets and metabolic abnormalities [2]. However, as our data show that mice expressing ligand-binding deficient VDR develop more severe skeletal defects than VDR-null mice, and that only the latter have hair defects, it will important to determine whether it is also the case in VDDR-II patients, to improve the diagnosis and treatment.

The possibility to restore the transcriptional activity of VDR mutants that are unresponsive to natural ligands is instrumental to identify VDR target genes. However, amongst various VDR agonist tested, none induced VDR^L233S^ transcriptional activity (our unpublished data). In contrast, we have shown that VDRgem transcriptional activity is efficiently induced by gemini ligands, and that they restore serum calcium levels of VDRgem mice, thus opening new avenues to characterize gene networks controlled by VDR ligands in the mouse [6]. Further phenotypic and molecular analyses of VDRgem, VDR-null and wild-type mice treated or not with 1,25(OH)_2_D_3_ or gemini ligands should allow to identify the signaling pathways controlled by unliganded and liganded VDR in various tissues, and thus facilitate the identification of new drug targets for various diseases, as well as VDR ligands with increased selectivity.
